# La rétinopathie de décompression oculaire: une complication rare de la trabéculectomie

**DOI:** 10.11604/pamj.2015.22.254.7483

**Published:** 2015-11-18

**Authors:** Ines Malek, Jihene Sayadi, Amine Masmoudi, Leila Nacef

**Affiliations:** 1 Service A d'Ophtalmologie, Institut Hedi Rais d'Ophtalmologie, Faculté de Médecine de Tunis, Université El Manar, Tunis, Tunisie

**Keywords:** Rétinopathie, décompression oculaire, trabéculectomie, retinopathy, eye decompression, trabeculectomy

## Abstract

Une patiente âgée de 50 ans, monophtalme de l’œil droit nous a été adressée pour une crise aigüe de glaucome par fermeture de l'angle de l’œil droit qui a évolué vers la chronicité malgré le traitement médical précoce. A j1 post trabéculectomie l'examen retrouve une hypotonie à 6 mmHg avec au du fond d’œil présence d'un œdème papillaire et de multiples hémorragies pré-rétiniennes rondes dont certaines à centre blanc localisées au pôle postérieur et en moyenne périphérie, épargnant la macula. L’évolution spontanée était favorable avec stabilisation de la pression intra-oculaire (PIO) à 12 mm Hg et nettoyage du fond de l’œil au bout de 6 semaines. Le bilan hématologique était sans particularités. La rétinopathie de décompression oculaire est une complication rare de la trabéculectomie. Son évolution est habituellement favorable. Dans certains cas une perturbation du bilan hématologique a été incriminée. Enfin cette complication peut être prévenue en évitant les variations brutales de la PIO.

## Introduction

La rétinopathie de décompression oculaire est une complication rare qui peut survenir après chirurgie filtrante du glaucome. Elle a été décrite pour la première fois par Fechtner et al en 1992 comme une rétinopathie hémorragique multifocale secondaire à une baisse brutale de la pression intraoculaire (PIO) et non expliquée par un autre processus [1]. Habituellement l’évolution est bénigne et spontanément résolutive [1, 2]. Cependant la possibilité d′un retentissement visuel permanent a été rapportée [2] d′où l′importance de reconnaître et de prévenir cette entité. Nous rapportons le cas d′une patiente opérée de trabéculectomie et qui a présenté dans les suites opératoires immédiates une rétinopathie de décompression oculaire.

## Patient et observation

Une patiente âgée de 50 ans, emmétrope a été adressée à nos urgences pour crise aigüe de glaucome par fermeture de l′angle (GFA) de l′œil droit. La patiente était monophtalme de l′œil droit, elle avait présenté 2 ans auparavant une crise de GFA de l′œil gauche qui a évolué vers un GFA chronique puis absolu malgré la trabéculectomie. Elle avait également bénéficié d′une iridotomie périphérique de l′œil droit. A l'admission, l′acuité visuelle (AV) de l′œil droit était limitée à la perception lumineuse. L′examen à la lampe à fente avait montré un cercle périkératique, une buée épithéliale, une hypothalamie et une semi mydriase aréflective. La PIO était à 54 mmHg à l'aplanation. L′examen du fond d′œil (FO) était inaccessible. La longueur axiale de l′œil droit était de 22,12 mm. L'examen de l’œil gauche avait trouvé: une AV nulle avec une PIO à 12 mmHg, une bulle de filtration fonctionnelle. La gonioscopie avait trouvé une fermeture synéchiante de l'angle et le FO avait montré une excavation totale.

**Figure 1:**
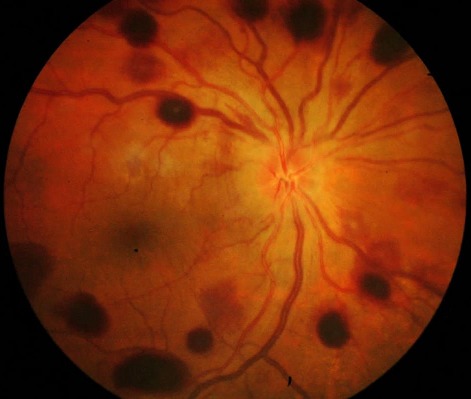
Photographie du fond d’œil droit montrant de multiples hémorragies pré-rétiniennes rondes à centre blanc localisées au pôle postérieur et en moyenne périphérie et un œdème papillaire

**Figure 2:**
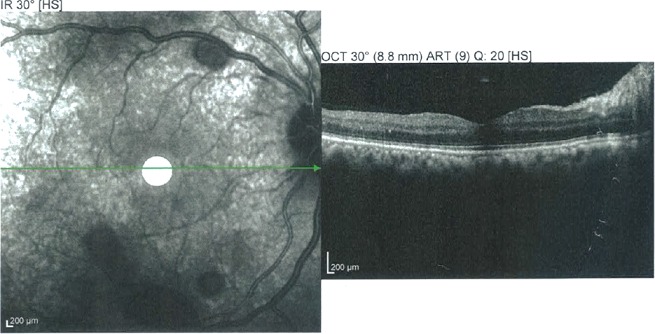
OCT passant par la macula de l’œil droit: épaisseur maculaire conservée, pas de décollement séreux rétinien

**Figure 3:**
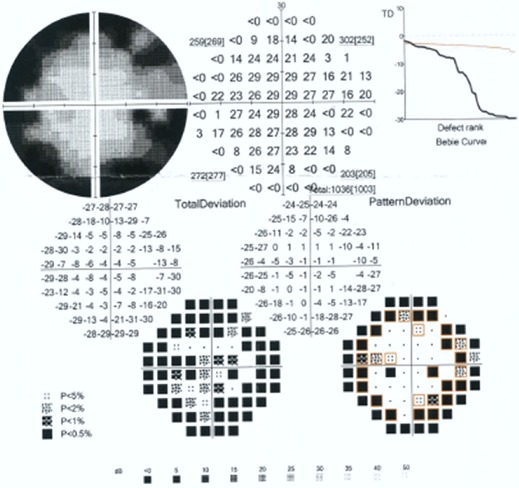
Champ visuel de l’œil droit montrant un scotome arciforme supérieur et inférieur au niveau de l'aire de Bjerrum

Un traitement médical urgent hypotonisant a été instauré. L’évolution a été marquée par la persistance d'une hypertonie à 35 mmHg à l’œil droit malgré le traitement médical maximal. L'AV était à 4/10 et la gonioscopie dynamique avait mis en évidence des synéchies antérieures périphériques étendues. Le diagnostic de glaucome chronique à angle fermé de l’œil droit était retenu, la patiente a bénéficié d'une trabéculectomie. A j1 postopératoire, l′examen avait trouvé une AV à 1/10, une bulle de filtration saillante, un seidel négatif, une chambre antérieure étroite, une PIO à 6 mmHg. L′examen du FO avait noté la présence d′un œdème papillaire et de multiples hémorragies pré-rétiniennes rondes, localisées au pôle postérieur et en moyenne périphérie de 0,5 à 2 diamètres papillaires, dont certaines à centre blanc, épargnant la macula et associées à des hémorragies péripapillaires superficielles sans décollement choroïdien par ailleurs (Figure 1). L'OCT était sans anomalies ne montrant notamment pas d’œdème maculaire ni de décollement séreux rétinien (Figure 2). L’évolution spontanée était favorable avec amélioration de l'AV à 4/10, stabilisation de la PIO à 12mmHg et nettoyage du FO au bout de 6 semaines. Le champ visuel avait montré des scotomes arciformes au niveau de l'aire de Bjerrum (Figure 3). Le bilan hématologique était sans particularités.

## Discussion

La rétinopathie de décompression est un terme utilisé dans la littérature pour désigner deux entités cliniques différentes: le syndrome de décompression aigue appelé aussi caisson ou maladie des plongeurs [3, 4] et la rétinopathie de décompression oculaire secondaire à une baisse brutale de la PIO et non expliquée par un autre processus [5]. Suzuki et al ont décrit trois types de rétinopathie hémorragiques qui peuvent survenir après chirurgie filtrante du glaucome: (1) Petites hémorragies de la périphérie rétinienne. (2) Hémorragies massives mais bénignes. (3) Occlusion de la veine centrale de la rétine qui peut être responsable d′un retentissement visuel important [6]. Près de la moitié des cas de rétinopathie de décompression oculaire rapportés dans la littérature ont fait suite à une trabéculectomie [5, 7]. Néanmoins cette entité peut aussi survenir suite à une insertion d′une valve de drainage [8], une sclérectomie profonde non perforante [9], une trabéculotomie [10], une iridotomie périphérique [11], une iridoplastie [12], une ponction chambre antérieure, une décompression orbitaire, une phacoémulsification [7], une vitrectomie et même à un traitement médical hypotonisant.

Une PIO élevée en préopératoire et une variation importante de la pression intraoculaire sont des facteurs de risque de la rétinopathie de décompression oculaire [13]. Les anomalies hématologiques préexistantes (Anémie, hémodilution, allongement du temps de prothrombine et du temps de la thromboplastine partielle activée) seraient également des facteurs favorisant la survenue de cette complication [13]. La physiopathologie de la rétinopathie de décompression oculaire est encore méconnue. La théorie mécanique serait la plus admise. Elle stipule qu′une baisse brutale de la PIO peut être à l'origine d'un déplacement antérieur et une expansion de la lame criblée. Ce mouvement diminuerait le flux axoplasmique, aboutissant à un œdème papillaire lequel comprime la veine centrale de la rétine d′où les hémorragies rétiniennes diffuses [7, 11, 12, 14]. Cliniquement, la rétinopathie de décompression oculaire est caractérisée par: des hémorragies rétiniennes diffuses, un œdème papillaire, un œdème maculaire et une baisse de l′acuité visuelle. Ces quatre signes sont exceptionnellement réunis [15]. Les hémorragies peuvent toucher toutes les couches rétiniennes mais sont principalement intra-rétiniennes. Elles se localisent surtout au niveau du pôle postérieur et en péripapillaire [5]. Des hémorragies à centre blanc sont observées dans 20% des cas [2, 5]. L′œdème maculaire et les décollements séreux rétiniens ont été exceptionnellement rapportés [14, 15].

L′évolution est spontanément résolutive au bout de 13 semaines en moyenne [5]. Cependant certains patients ont nécessité une vitrectomie pour une hémorragie intravitréenne ou rétro hyaloïdienne sans tendance à la résorption [5]. La prévention de la rétinopathie de décompression passe par un abaissement progressif de la pression intraoculaire en pré et per-opératoire et le dépistage des patients à risque notamment ceux qui présentent des troubles hématologiques [13].

## Conclusion

La rétinopathie de décompression oculaire est une complication rare de la trabéculectomie qui se voit chez les patients présentant une forte hypertonie en préopératoire et une hypotonie marquée en postopératoire. Son évolution est habituellement favorable.
